# Conditional Most-Correlated Distribution-Based Load-Balancing Scheme for Hybrid LiFi/WiGig Network

**DOI:** 10.3390/s24010220

**Published:** 2023-12-30

**Authors:** Mohammed Farrag, Abdulrahman Al Ayidh, Hany S. Hussein

**Affiliations:** 1 Electrical Engineering Department, King Khalid University (KKU), Abha 62529, Saudi Arabia; aalaid@kku.edu.sa (A.A.A.); hany.hussein@aswu.edu.eg (H.S.H.); 2 Electrical Engineering Department, Assiut University, Assiut 71515, Egypt; 3 Electrical Engineering Department, Aswan University, Aswan 81528, Egypt

**Keywords:** load balancing, LiFi communications, WiGig applications, hybrid LiFi/RF network

## Abstract

A hybrid network has recently been proposed as a framework for a high-speed wireless communication network. Basically, it integrates light fidelity (LiFi) with radio frequency wireless gigabit alliance (WiGig) networks that operate, simultaneously, in a completely different frequency band. To assign the best access point (AP) and provide enough resources for each user, an effective load-balancing (LB) strategy is needed. However, the traditional LB strategies involve sophisticated iterative computing procedures whenever the user distribution changes. Hence, the first contribution of this work is to offer a more adaptable, two-step, conditional, and most-correlated distribution (CMCD) algorithm. Thus, the low-complexity most-correlated distribution (MCD) LB scheme is applied, and the average data rates for all users are then calculated. If the results achieve the predefined performance threshold (PDT), the decisions will be confirmed; otherwise, the proposed scheme automatically switches to the more accurate, but more complex, consecutive assign WiGig first separate optimization algorithms (CAWFS) algorithm. The suggested algorithm provides a clear performance-complexity trade-off, which could be simply controlled by choosing the suitable performance tolerance factor. The second contribution of this paper is the correlation-weighted majority voting (CWMV) method, which attempts to benefit from as many prior decision votes as possible, instead of relying just on one vote. In the CWMV technique, the weight of each vote is calculated based on the correlation between the history distribution vectors and the new user distribution vector. A significant increase in the system performance is evident from the simulation results.

## 1. Introduction

Mobile communication networks are now operating at full capacity because of the restricted radio frequency (RF) spectrum and the increasing number of mobile devices with data-demanding applications and multimedia materials (i.e., images, audio, online gaming and animation, video streaming, etc.). As a possible remedy for the spectrum scarcity issue, novel light fidelity (LiFi) technology is suggested [1,2,3,4]. LiFi runs in the 300 THz vacant and free-licensed optical zone. LiFi access points (APs) offer connectivity within a coverage range of a few square meters known as LiFi attocells. Due to their tiny size, the attocells produce excellent spatial-spectral efficiency (SE) and encourage frequency reuse by preventing adjacent LiFi AP users from inter-cell interference [5]. In [6], research on the next-generation optical communication infrastructure—evolving concurrently with the demands of mobile communication systems requiring 5G and beyond—was presented. This research included optical-wireless communication (OWC), free-space optical communication (FSOC), and visible-light communication (VLC). In [7], they justified the continued push to develop optical access networks. They outlined the difficulties that are increased due to traffic dynamicity and heterogeneity as well as the computational resource limitations of these networks. They then presented the cutting-edge machine-learning techniques being investigated to deal with these issues. As the number of mobile device users increases, the LiFi networks are faced with many technical challenges. User mobility and related difficulties, including the obstruction of users’ light path, handover, and imperfectly oriented connections, is one of these challenges [8]. Furthermore, employing visible light for the uplink could distract mobile users. Also, simultaneous communication could not be established because of uplink and downlink interference [9].

In a different direction, in order to solve the issue of spectrum scarcity, the WiGig technology provides a potential RF communication architecture that makes use of a new WiFi protocol with an extraordinarily high millimeter-wave (mm-wave) transmission capacity [10]. In [11,12], for a satellite-terrestrial integrated network, where a multibeam satellite system shares the mm-wave spectrum with a cellular system, they examined the secrecy-energy-efficient hybrid beamforming (BF) techniques which aimed to optimize the feasible energy-secrecy efficiency while meeting the cellular customers’ and earth stations’ SINR requirements. However, when the number of served users increases over a specific threshold, side-lobe levels also increase, which raises the inter-beam interference (IBI) [13,14]. Therefore, in order to optimize the SE, it is suggested that just a small number of users be supported [15]. These problems may be solved, and the quality of service (QoS) can be improved using the hybrid LiFi/RF network, as suggested in [16,17,18].

A hybrid network that combines both LiFi and WiGig APs is feasible because LiFi and WiGig operate in separate frequency bands. This ensures that their applications will not interfere with each other [8,19,20,21]. A single light-emitting diode (LED) can produce data at a rate of more than 3 Gb/s, whereas WiGig AP offers a data throughput of 7 Gb/s [17,18]. So, the hybrid LiFi/WiGig network performs better than a standalone LiFi or WiGig system [21]. Furthermore, in order to serve use cases like eMBB (enhanced mobile broadband), integration networks for RF and LiFi will be required for the next 6G network [22]. One of 6G’s main use cases, eMBB, aims to increase the capacity, speed, and coverage of mobile broadband networks. This may be done by permitting the combination of mm-waves and LiFi, which increases the demand for a reliable load-balancing (LB) system.

Only one AP—LiFi or WiGig—should be available to each user on the hybrid network. To ensure optimal user throughput, stability, and fairness, a load-balancing (LB) technique is required. The LB technique consists of two key procedures: access point assignment (APA) and resource allocation (RA) [23]. Joint load-balancing and power allocation solutions were recommended for hybrid RF/visible light communication (VLC) networks in [24]. An iterative strategy has been established to raise the overall system capacity and enhance the system fairness. The authors in [25] proposed an APA framework that uses the multi-criteria decision-making (MCDM) method for users in a hybrid LiFi/WiFi network. A comparison of LB schemes is shown in [26], with examples including the fuzzy logic-based scheme (FBS) and the joint and separate optimization algorithms (JOA and SOA, respectively). In terms of the user data rate, the simulation research shows that JOA performs significantly better than SOA and becomes close to the global optimum. The computational complexity of SOA is, nevertheless, far lower than that of JOA. The fundamental issue with all of the LB algorithms that was previously stated is that they were all created for the conventional WiFi scheme as a representative of RF technology, but not for the proposed WiGig. Therefore, they neglected to consider the strict limit on the maximum number of mobile users that may be allocated to the WiGig AP [15].

According to [21], two modified versions of the SOA algorithm—the assign WiGig first SOA (AWFS) algorithm and the consecutive assign WiGig first SOA (CAWFS) methods—were created to address this issue. With the new algorithms, the WiGig AP is only allocated to $N_{max}$ users with a minimum LiFi data rate, where $N_{max}$ is the maximum number of mobile users that can be allocated to the WiGig AP. The simulation results showed that the two proposed algorithms performed better than the SOA strategy in terms of practical data rates and outage probability. However, unfortunately, the computational complexity of the proposed techniques is much higher than that of SOA.

This issue was addressed in [27] with the most-correlated distribution (MCD)-based LB scheme. With reduced computational complexity compared to existing LB methods, this technique sought to provide equivalent feasible data rate and outage probability characteristics. The MCD algorithm’s fundamental concept was not to repeat the APA optimization computations for each user’s distribution. Instead, to determine the best decisions for the new distribution of mobile users, the MCD algorithm used the history of all feasible distributions of mobile users and the related APA decisions, which were kept in a distributions-decisions record (DDR). Depending on any LB scheme, the CPU unit created the DDR once and offline. Without loss of generality, the CAWFS method was used in this study to create the DDR record [21]. The expected distribution and the corresponding APA decisions made up each row in the DDR record. The DDR record subset that most closely correlates with the new distribution of mobile users was created. The current decisions are decided based on the previous decisions made in the defined subset using the majority voting technique. In order to lower the total computing complexity, the MCD presented a unique, straightforward technique that may be used based on any current LB scheme. However, there is no guarantee in the MCD algorithm that the selected APA choices will provide a sufficient data rate and/or outage probability performance.

The first contribution of this paper is to propose a more adaptable, two-step, conditional most-correlated distribution (CMCD) algorithm. The low-complexity MCD algorithm [27] is used in the first step to make the APA choices, and the average data rate performances for users allocated to LiFi and WiGig APs, $R_{l}$ and $R_{w}$, respectively, are calculated. If the chosen APA achieves the predefined performance threshold $\eta_{th}$, the decisions will be confirmed, and the DDR record will be updated to include the new distribution and its related APA choices. The second step will begin to calculate the APA decisions using the more accurate but more complex CAWFS algorithm [21] if, on the other hand, the given performance is below the $\eta_{th}$ threshold. The DDR is then supplemented with the CAWFS-calculated distribution decisions. The proposed CMCD method’s relative complexity will depend on how frequently the CAWFS algorithm is invoked. The CMCD algorithm complexity will be at its lowest level and the MCD algorithm performance is supplied when the MCD algorithm is completely used and no calls to the CAWFS algorithm are needed. On the other hand, when the MCD algorithm fails to supply the required performance and the CAWFS algorithm is completely activated, the CMCD algorithm complexity will increase to its maximum level and the CAWFS algorithm performance will be provided.

The second contribution is the correlation-weighted majority voting (CWMV) scheme. The main objective of the majority voting scheme is to profit from the largest number of preceding decision votes rather than depending on only one associated vote. In the previous work [27], the majority vote outcome for each column in the voting matrix $V_{T}$ is the decision with the highest likelihood of repetition. The flaw with this system is that it treats all of the voting column’s components equally, regardless of how closely their respective distribution vectors correlate with the incoming distribution vector. To solve this problem in the proposed CWMV scheme, the voting column’s components are weighted by the correlation values between their respective distribution vectors and the incoming distribution vector.

The remainder of this work is structured as follows: The channel models for the LiFi and WiGig sub-networks as well as the hybrid system model, are presented in Section 2. The previously suggested SOA, CAWFS, and MCD algorithms are reviewed in Section 3. An extensive discussion of the proposed CMCD LB algorithm is provided in Section 4. The simulation and discussion of the throughput analysis and performance evaluation are presented in Section 5. This paper concludes with Section 6.

## 2. The Hybrid System and Channel Models of the LiFi and WiGig Sub-Networks

### 2.1. System Model

**Figure 1:**
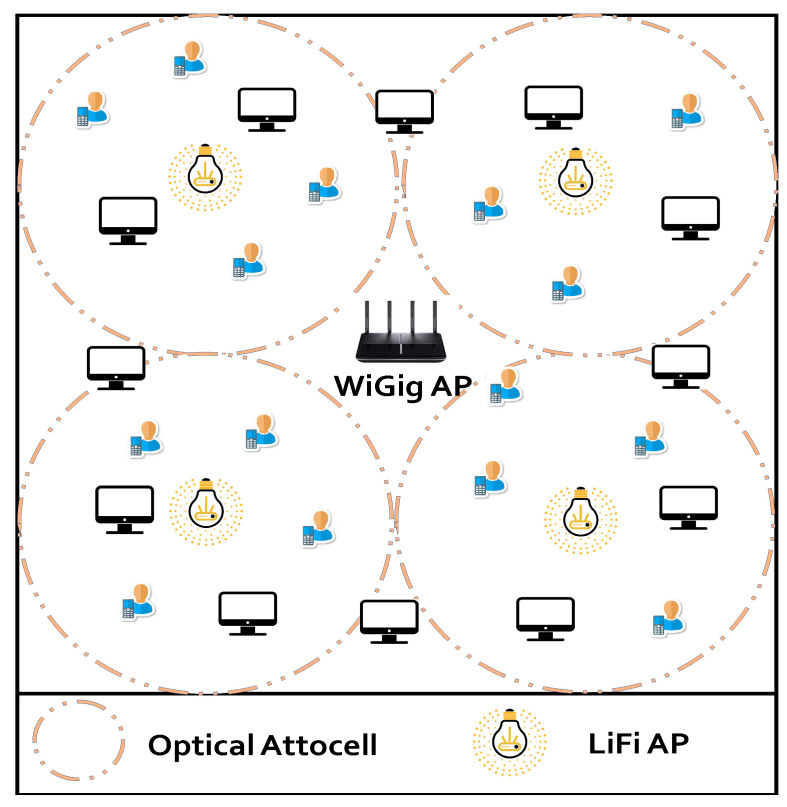
The proposed system model.

One WiGig AP and many $N_{LF}$ LiFi APs are distributed throughout the coverage area’s ceiling in the recommended system, which is shown in Figure 1. $U={\left\{\mu_{i}\right\}}_{i=1}^{N_{\mu }}$, a group of $N_{\mu }$ users, are dispersed in a partially random distribution (PRD) model around the space. While some users are free to roam to any position within the covered area, the other users in the PRD are partially tethered, within 0.5 m of particular fixed spots to represent people seated around fixed tables or offices. The considered system utilizes error-free communication to connect a central processing unit (CPU) to each AP.

The AP of the LiFi sub-network, composed of many light-emitting diodes (LEDs), is coupled to mobile devices through photodetectors (PDs) with identical irradiance and incidence angles. Due to the fact that all LiFi APs reuse the spectrum frequency, the LiFi system has exceptional spatial efficiency (SE) [28]. If a user moves in the overlapping area between neighboring cells, inter-carrier interference (ICI), which can lower the user throughput, may happen [16,29]. This issue may be solved by increasing system throughput with a WiGig AP. For downlink communication, each mobile user is allocated to only one LiFi or WiGig AP. Both the time slot resource allocation (RA) and access point assignment (APA) tasks must be managed by the network LB system. The APA and RA processes should be updated for each quasi-static state $T_{n}$ in the dynamic indoor scenario, where *n* is the state sequence number [29]. In the hypothetical system, the LiFi and WiGig access points are denoted by $C=\{c|c\in \left[0,N_{LF}\right],c\in Z\}$, where $\left(c=0\right)\in C_{R}$ denotes the WiGig AP, $C_{l}={\left\{c\right\}}_{l=1}^{N_{LF}}$ denotes the LiFi APs, and Z denotes the set of integer numbers.

### 2.2. Model for the LiFi Channel

In environments of indoor communication, line of sight (LoS) and reflection make up the two main sections of the optical channel’s gain. When utilizing LiFi with a baseband modulation bandwidth (B) smaller than 25 MHz, it is possible to ignore the reflection component [29]. Furthermore, the LoS component represents at least 95% of the total energy collected by LiFi PDs [30,31]. Therefore, in our studied model of the LiFi channel, the reflection component will be disregarded given that the bandwidth B=20 MHz is considered. The authors in [32] define the LoS component as follows:

(1) $$H_{\mu ,\alpha }=\left\{\begin{array}{cc} \frac{\left(m+1\right)A_{p}g\left(\theta \right)T_{s}\left(\theta \right)}{2\pi (z^{2}+\omega^{2})}\cos^{m}\left(\phi \right)\cos\left(\theta \right), & \;0\leq \theta \leq \Theta_{F}\\ 0, & \;\theta <\Theta_{F} \end{array}\right.$$

where $m=-1/\log_{2}(\cos\left(\theta_{1/2}\right)$ is the Lambertian index with $\theta_{1/2}$ which is the half-intensity radiation angle; $A_{p}$ is the photo-detector physical area; *z* is the horizontal distance between the mobile user and the LiFi AP $\alpha^{th}$; ω is the room height; θ and ϕ are the incidence and irradiation angles, respectively; $\Theta_{F}$ is the receivers half angle of the filed-of-view (FOV); $T_{s}\left(\theta \right)$ is the optical filter gain; and g(θ) is the gain concentrator which is defined as [32]:

(2) $$g\left(\theta \right)=\left\{\begin{array}{cc} \frac{\chi^{2}}{\sin^{2}\left(\Theta_{F}\right)}, & \;0\leq \theta \leq \Theta_{F}\\ 0, & \;\theta <\Theta_{F} \end{array}\right.$$

with the refractive index χ.

In order to send LiFi signals in optical power form, baseband communication employing intensity modulation (IM) and direct detection (DD) is employed in LiFi systems [33]. The average DC optical power $P_{opt}$ and the average electric power of signals $P_{elec}$ are related as follows [34]:

(3) $$\iota =P_{opt}/\sqrt{P_{elec}}$$

For a mobile user μ, that is assigned to α AP, the signal-to-interference-plus-noise ratio (SINR) is [32]:

(4) $${\mathrm{SIN}R}_{\mu ,\alpha }=\frac{{\left(\kappa P_{opt}H_{\mu ,\alpha }\right)}^{2}}{\iota^{2}N_{0}B+{\left(\kappa P_{opt}\right)}^{2}\sum H_{\mu ,else}^{2}}$$

where κ is the receivers’ optical-to-electric conversion efficiency; $N_{0}\left[A^{2}/\mathrm{Hz}\right]$ is the noise power spectral density; $H_{\mu ,\alpha }$ is the channel gain between the $\alpha^{th}$ LiFi AP and the $\mu^{th}$ mobile user; and $H_{\mu ,else}$ is the channel gain between the interfering LiFi APs and the same user, using Equation (1). The Shannon capacity is used to determine the maximum data rate that may be achieved between the designated mobile user μ and the LiFi AP α as follows:

(5) $$R_{\mu ,\alpha }^{\left(n\right)}=B\log_{2}\left(1+{\mathrm{SIN}R}_{\mu ,\alpha }^{\left(n\right)}\right),$$

### 2.3. Model for the WiGig Channel

The proposed WiGig communication sub-network consists of only one WiGig AP and $N_{\mu }\leq N_{max}$ simultaneous mobile users [15]. The WiGig AP has $N_{BS}$ antennas and $N_{RF}$ RF chains. Through a single stream, each mobile user μ is linked to the WiGig AP with $N_{MS}$ antennas. In the downlink, the transmitted signal, for *U* mobile users, is represented by [35]

(6) $$x=F_{RF}F_{BB}s,$$

where $F_{BB}$ is a U×U baseband pr-encoder; $F_{RF}$ is an $N_{BS}\times U$ RF pr-encoder; and s is the U×1 transmitted symbols vector with $E\left[ss^{*}\right]=\left(P/U\right)I_{U}$, and average total transmitted power *P*.

The received signal at the $\mu^{th}$ user could be assumed in the narrow-band block-fading channel model described in [14,15,35,36] which might be written as:

(7) $$r_{\mu }=H_{\mu }\overset{U}{\sum_{n=1}}F_{RF}f_{n}^{BB}s_{n}+n_{\mu }$$

where $H_{\mu }$ is the mm-wave channel between the $\mu^{th}$ user and the WiGig AP, and the receiver’s signal is being distorted by $n_{\mu }∼N\left(0,\sigma^{2}I\right)$ Gaussian noise. At the $\mu^{th}$ user, the received signal $r_{\mu }$ is addressed by the RF combiner $w_{\mu }$ as follows:

(8) $$y_{\mu }=w_{\mu }^{*}H_{\mu }\overset{U}{\sum_{n=1}}F_{RF}f_{n}^{BB}s_{n}+w_{\mu }^{*}n_{\mu }$$

In [35], a geometric channel model with $L_{\mu }$ scatterers for the user μ’s channel to account for the predicted low scattering in the mm-wave channel is used. Every scatterer represented a separate propagation path from the user μ to the BS. In [37], a straightforward geometric explanation of the scattering environment and an intermediate virtual channel model that represents physical modeling without getting bogged down in details were given. In this paradigm, the $H_{\mu }$ channel may be written as follows:

(9) $$H_{\mu }=\sqrt{\frac{N_{BS}N_{MS}}{L_{\mu }}}\overset{L_{\mu }}{\sum_{l=1}}\rho_{\mu ,l}a_{MS}\left(\theta_{\mu ,l}\right)a_{BS}^{*}\left(\phi_{\mu ,l}\right),$$

where $\rho_{\mu ,l}$ is the $l^{th}$ path complex gain, with $E[|\rho_{\mu ,l}{|}^{2}]=\bar{\rho }$. $\theta_{\mu ,l}$ and $\phi_{\mu ,l}\in \left[0,2\pi \right]$ are the $l^{th}$ path’s angles of arrival and departure (AoAs/AoDs), respectively. Finally, $a_{MS}\left(\theta_{\mu ,l}\right)$ and $a_{BS}^{*}\left(\phi_{\mu ,l}\right)$ are the vectors response of the antenna array for the WiGig AP and $\mu^{th}$ user, respectively. Then, the achievable rate at the μ’s user could be represented by [35]:

(10) $$R_{\mu }=\log_{2}\left(1+\frac{\frac{P}{U}{\left|w_{\mu }^{*}H_{\mu }F_{RF}f_{\mu }^{BB}\right|}^{2}}{\frac{P}{U}\sum_{n\neq \mu }{\left|w_{\mu }^{*}H_{\mu }F_{RF}f_{n}^{BB}\right|}^{2}+\sigma^{2}}\right).$$

Therefore, the system’s sum-rate is:

(11) $$R_{sum}=\overset{N_{max}}{\sum_{\mu =1}}R_{\mu }.$$

## 3. Previously Proposed LB Algorithms

In this section, a review of a group of previously proposed algorithms is provided.

### 3.1. Separate Optimization Algorithm (SOA)

In the SOA algorithm, the RA and APA processes are successively optimized [26]. In order to increase the spatial SE of the LiFi sub-network, the users whose LiFi data rates surpass a specific threshold level γ will be allocated to LiFi APs in the APA process. Nevertheless, the RF APs will be given to the other users. Additional criteria, such as the maximum effective throughput, are applied. For the $\mu^{th}$ user, consider that:

(12) $$r_{\mu ,c}=\left\{\begin{array}{cc} R_{\mu ,\alpha }, & \mathrm{if}\;c\in C_{L}\\ R_{\mu }, & \mathrm{if}\;c\in C_{R} \end{array}\right.$$

where $R_{\mu ,\alpha }$ and $R_{\mu }$ will be calculated from Equations (5) and (10), respectively.

The selected LiFi AP with the maximum connection data rate is presented in [26] as follows:

(13) $$\tau_{1,\mu }=\arg\max_{{}_{j\in C_{L}}}r_{\mu ,j},$$

where rμ,j is the LiFi data rate. If every user in the LiFi attocell evenly shares the time resources, the possible data rate of each user is:

(14) $$\lambda_{\mu }=r_{\mu ,j}/M_{\tau_{1,\mu }}$$

where $M_{\tau_{1,\mu }}$ is the number of users assigned to the LiFi AP $\tau_{1,\mu }$. To assign users with $\lambda_{\mu }<\gamma$ to RF APs, the following criteria will be employed [26]:

(15) $$\begin{array}{c} \tau_{2,\mu }=\arg\max_{{}_{j\in C_{R}}}r_{\mu ,j},\;\;\lambda_{\mu }<\gamma . \end{array}$$

According to Equations (13) and (15), the APA result in SOA is:

(16) $$g_{\mu ,\alpha }^{\left(\mathrm{SOA}\right)}=\left\{\begin{array}{cc} 1, & \;\alpha =\left\{\begin{array}{cc} \tau_{1,\mu }, & \;\lambda_{\mu }\geq \gamma \\ \tau_{2,\mu }, & \;\lambda_{\mu }<\gamma \end{array}\right.\\ 0, & \;\mathrm{Otherwise} \end{array}\right.$$

During the RA stage, each AP gives the participants their own time allotment. It is possible to formulate the utility maximization problem that takes into consideration both user fairness and the sum-rate using the generalized β-proportional fairness function $\Psi_{\beta }\left(x\right)$ [38], where

(17) $$\Psi_{\beta }\left(x\right)=\left\{\begin{array}{cc} \log(x), & \;\beta =1\\ \frac{x^{1-\beta }}{1-\beta }, & \;\beta \geq 0,\beta \neq 1 \end{array}\right.$$

where *x* is the achievable data rate and β is the fairness coefficient. An explanation of the RA stage follows:

(18) $$k_{\mu ,\alpha }^{\left(\mathrm{SOA}\right)}=\frac{r_{\mu ,\alpha }^{\frac{1}{\beta }-1}}{\sum_{i\in U_{\alpha }}r_{i,\alpha }^{\frac{1}{\beta }-1}}\;\left(\beta >0\right).$$

In Algorithm 1, the steps of SOA are summarized.   
**Algorithm 1:** The SOA algorithm.
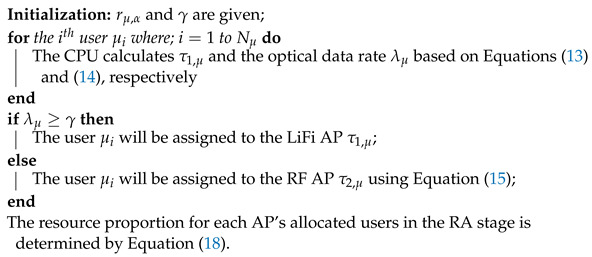


The performance of the SOA algorithm will be considerably impacted by the chosen threshold γ. The random distribution of the mobile users may result in a fairly high number of users being allocated to a particular LiFi AP $M_{\tau_{1,\mu }}$. Due to the LiFi APs’ resource constraints, the possible data rate $\lambda_{\mu }$ in Equation (14) will be less than the acceptable threshold. $\lambda_{\mu }$ will very certainly be smaller than γ if γ is slightly beyond the threshold. As a result, all of these users will share the RF AP, leaving the LiFi AP vacant and under-utilizing the network resources. Because of this, the SOA method cannot be employed with WiGig technology as the RF representation. There will be no consideration in this case for the rigorous cap on the maximum number of mobile users that may be assigned to the WiGig AP. The performance will consequently suffer and the inter-beam interference (IBI) will increase according to [15]. If the threshold γ is set to a very low value, users will not be allocated to the RF AP at all, or they will be allocated in very small numbers, which is another form of resource under-utilization. In the CAWFS algorithm (Section 3.2), this issue could be resolved.

### 3.2. Consecutive Assign WiGig First SOA (CAWFS) LB Algorithm

Through the usage of the CAWFS algorithm, each of the $N_{max}$ users is assigned, one by one, to a WiGig AP. Initially, all users are grouped into a set M. Based on Equation (13), each user is allocated to the best LiFi AP. Using Equation (14), identify $\mu_{min}$; the user with the lowest possible LiFi rate. This user will be allocated to the WiGig AP and removed from M set. Distributing the remaining $M=M-\mu_{min}$ users among all LiFi APs based on Equation (1), then, the new potential $\gamma_{\mu }$ is determined. The procedure above is repeated up until $N_{max}$ users—the permitted maximum—have been assigned to the WiGig AP. Possible displays for the APA output of the CAWFS algorithm are as follows:

(19) $$g_{\mu ,\alpha }^{\left(\mathrm{CAWFS}\right)}=\left\{\begin{array}{cc} 1, & \;\alpha =\left\{\begin{array}{cc} \tau_{1,\mu }, & \;\mu \in M\\ \mathrm{WiGig}\;\mathrm{AP}, & \;\mu \notin M \end{array}\right.\\ 0, & \;\mathrm{Otherwise} \end{array}\right.$$

The CAWFS steps are outlined in Algorithm 2.   
**Algorithm 2:** The CAWFS algorithm
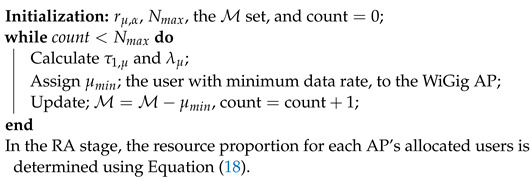


### 3.3. MCD Load-Balancing Algorithm

The MCD algorithm was proposed in [27] to provide an appropriate performance/ complexity trade-off. Both considerable system throughput and a reduction in significant computational complexity are possible with the MCD approach. The main tactic of the suggested technique is to avoid repeating the APA computations again for every user’s distribution. In order to choose the best APA decisions for the new mobile users’ distribution, the MCD algorithm looks back at all potential distributions of mobile users and the related APA decisions that are archived in a distributions–decisions record (DDR).

There are two steps in the MCD system. The most common case of users’ distributions and the best APA decisions that go along with them are compiled in the first step, known as the DDR construction stage. The assumed distribution from the distributions matrix DM and the corresponding decisions make up each raw in the DDR. The CPU builds the DDR once and offline. The decision of each user could be:

(20) $$D_{u}\in {\left\{D_{i}\right\}}_{\left(i=0\right)}^{\left(N_{LF}\right)}$$

for WiGig, AP i=0 is utilized, and other values for *i* are used for LiFi APs. Algorithm 3 displays the pseudo algorithm for the DDR construction procedure.


**Algorithm 3: The DDR construction algorithm in MCD**

*The CPU runs this algorithm once and offline*


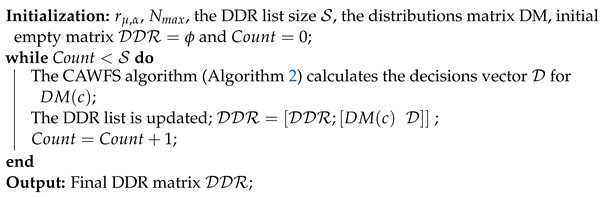



The current decisions are computed at the second decision selection (DS) stage based on the current distribution of mobile users in the $d_{u}$ vector depending on the modified majority voting technique [27]. The decision with the highest recurring probability, in each column in the voting matrix $V_{T}$, is the majority vote. Algorithm 4 displays the pseudo algorithm for the DS stage.   
**Algorithm 4:** The DS algorithm
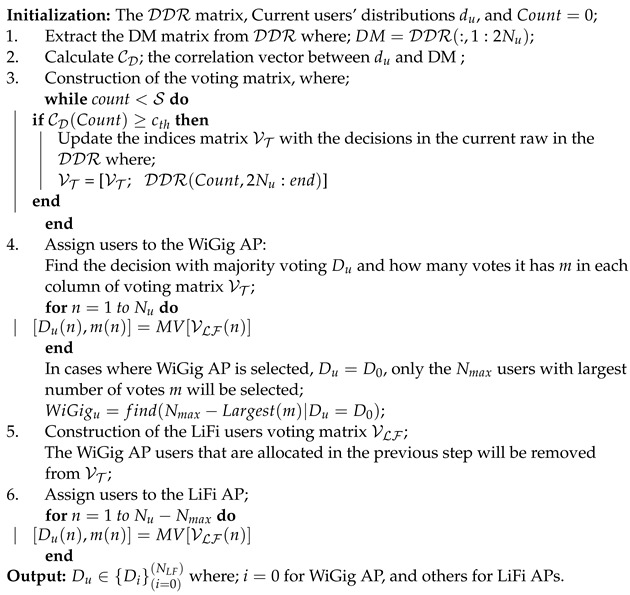


## 4. Proposed Conditional MCD-LB Scheme

Unfortunately, in the MCD algorithm [27], there is no assurance that the chosen APA decisions will offer a satisfactory data rate and/or outage probability performances. So, in this work, a more flexible LB scheme is proposed. The proposed conditional most correlated distribution (CMCD) algorithm is composed of two stages. In the first stage, the APA decision is taken based on the MCD algorithm from [27] and the average data rate performances of both LiFi and WiGig APs assigned users, $R_{l}$ and $R_{w}$, respectively, determined whether this decided APA provides acceptable performance. Compared to predefined threshold $\eta_{th}$, these decisions will be confirmed, and the DDR record will be updated by adding the new distribution and its corresponding APA decisions. On the other hand, if the provided performance is less than the threshold $\eta_{th}$, the second stage will be started to compute the APA decisions using the more complex but more accurate CAWFS algorithm. Then, the calculated distribution decisions, using CAWFS, are added to the DDR.

The comparable complexity of the proposed CMCD algorithm depends on how many times the CAWFS algorithm will be called. When the MCD algorithm is fully used and no calls to the CAWFS algorithm take place, the CMCD algorithm complexity will be at its minimum level and the MCD algorithm performance is provided. On the other hand, the CMCD algorithm complexity will reach its maximum level, and the CAWFS algorithm performance will be offered when the MCD algorithm fails to deliver an acceptable performance and the CAWFS algorithm is fully invoked. In the simulation section, if the CMCD algorithm is running for $N_{T}$ times, and the MCD algorithm succeeds in achieving an acceptable performance for $N_{MCD}$ times, the complexity reduction ratio CRR, compared with the CAWFS algorithm, will be defined as:

(21) $$\mathrm{CRR}=N_{MCD}/N_{T}$$

The minimum data rate for both LiFi and WiGig AP users $\lambda_{min}\left(LiFi\right)$ and $\lambda_{min}\left(WiGig\right)$, respectively, could be used as a calling threshold for the CAWFS algorithm. Therefore, it directly affects the overall system complexity reduction ratio CRR. In the proposed algorithm, the MCD algorithm uses a DDR record, which slightly differs from the one used in the conventional MCD algorithm and is shown in Algorithm 3. One more step to calculate both $\lambda_{min}\left(LiFi\right)$ and $\lambda_{min}\left(WiGig\right)$ is added, and the modified version is shown in Algorithm 5. The second modification in the MCD algorithm is using the correlation-weighted majority voting (CWMV) from Section Correlation-Weighted Majority Voting Scheme, instead of using the majority voting technique used with the previous algorithm in [27].   
**Algorithm 5:** The DDR construction algorithm in the proposed CMCD *The CPU runs this algorithm once and offline*
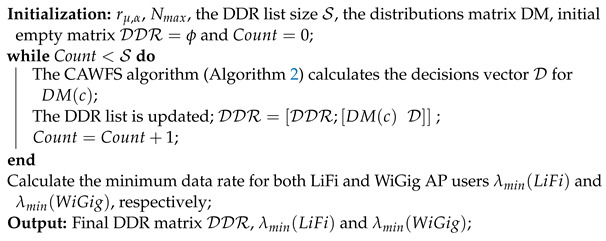


Algorithm 6 displays the pseudo algorithm for the DS stage in the suggested CMCD scheme.
**Algorithm 6:** The proposed CMCD algorithm**Initialization:** DDR, $d_{u}$, and $\eta_{th}$1.  Using DS in MCD algorithm (Algorithm 4) to find the decision vector D2.  Calculate the average data rate of LiFi and WiGig APs users, $R_{l}$ and $R_{w}$, respectively;3.  The output is compared with the threshold $\eta_{th}$;  **if** $\left\{\begin{array}{cc} R_{l}\geq \eta_{th}, & if\;the\;LiFi\;output\;is\;optimized.\\ & or\\ R_{w}\geq \eta_{th}, & if\;the\;WiGig\;output\;is\;optimized. \end{array}\right.$**then** | D is confirmed, and DDR is updated  **else** | More accurate D is calculated using CAWFS Section 3.2, and DDR is updated  **end****Output:** $D_{u}\in {\left\{D_{i}\right\}}_{\left(i=0\right)}^{\left(N_{LF}\right)}$ where; i=0 for WiGig AP, and others for LiFi APs;

### Correlation-Weighted Majority Voting Scheme

The primary goal of the majority voting scheme is to benefit from the greatest number of prior decision votes rather than relying on only one single correlated vote. In [27], the majority vote outcome for each column in the voting matrix $V_{T}$ is the decision with the highest likelihood of repetition. The flaw with this system is that it treats all of the voting column’s components equally, regardless of how closely their respective distribution vectors correlate with the incoming distribution vector. To solve this problem in the proposed correlation-weighted majority voting (CWMV) scheme, the voting column’s components are weighted by the correlation values between their respective distribution vectors and the incoming distribution vector. Given that the *n*th voting vector equals

(22) $$V_{T}\left(n\right)=\left[D\left(1\right)D\left(2\right)\ldots D\left(N_{\mu }\right)\right];D\left(\mu \right)\in {\left\{D_{i}\right\}}_{\left(i=0\right)}^{\left(N_{LF}\right)}$$

where D(μ) is the assigned AP for the $\mu^{th}$ user; i=0 for WiGig AP; and others for LiFi APs. Suppose that the correlation values between their respective distribution and the incoming distribution vectors equal $C_{V}$ where

(23) $$C_{V}=\left[\sigma \left(1\right)\sigma \left(2\right)\ldots \sigma \left(N_{\mu }\right)\right];$$

where σ(i) is the correlation value between the incoming distribution vector and the $i^{th}$ rows of the DDR su-set that satisfy the correlation threshold $c_{th}$ condition. Depending on those correlation values, the weight for decision D(i) is calculated as follows:

(24) $$\omega_{i}=\left\lfloor 10\times \sigma \left(i\right)\right\rfloor ;\mathrm{for}\;i=0\;\mathrm{to}\;N_{\mu }$$

where ⌊x⌋ is the greatest integer that is less than or equal to *x*. Let $1_{\omega i}=\left[11\ldots 1\right]$ be an all-one vector of length $\omega_{i}$. The new voting matrix $V_{N}\left(n\right)$ will be

(25) $$V_{N}\left(n\right)=\left[D\left(1\right)\times 1_{\omega 1}D\left(2\right)\times 1_{\omega 2}\ldots D\left(N_{\mu }\right)\times 1_{\omega N\mu }\right];$$

where the $i^{th}$ element of $V_{T}$ is repeated with number $\omega_{i}$. For example, if the decisions in $n^{th}$ voting column of $V_{T}$ equal $V_{T}\left(n\right)=\left[D_{1}D_{2}D_{2}D_{3}\right]$, and the correlation values between their respective distribution and the incoming distribution vectors equal $C_{V}=\left[0.6\;0.2\;0.1\;0.1\right]$, the majority voting using the previous algorithm, in [27], will be $D_{2}$; the decision with the highest probability in $V_{T}\left(n\right)$. If the proposed CWMV algorithm is applied, the weighting vector will be ω=[6211], and the majority voting will be calculated depending on the new vector $V_{N}\left(n\right)=\left[D_{1}D_{1}D_{1}D_{1}D_{1}D_{1}D_{2}D_{2}D_{2}D_{3}\right]$, where the $i^{th}$ element of $V_{T}$ is repeated with $i^{th}$ value in the weighted vector ω. So, the majority voting will be $D_{1}$.

## 5. Performance Evaluation

### 5.1. Simulation Setup

In this simulation section, the same system configuration and simulation parameters from [21,23,29,39,40] are used. One WiGig AP and four LiFi APs make up the hybrid network that is being considered. The value of $N_{max}=6$ users is selected in this study, where the best throughput is offered based on the simulation findings in [21,27].

**Figure 2:**
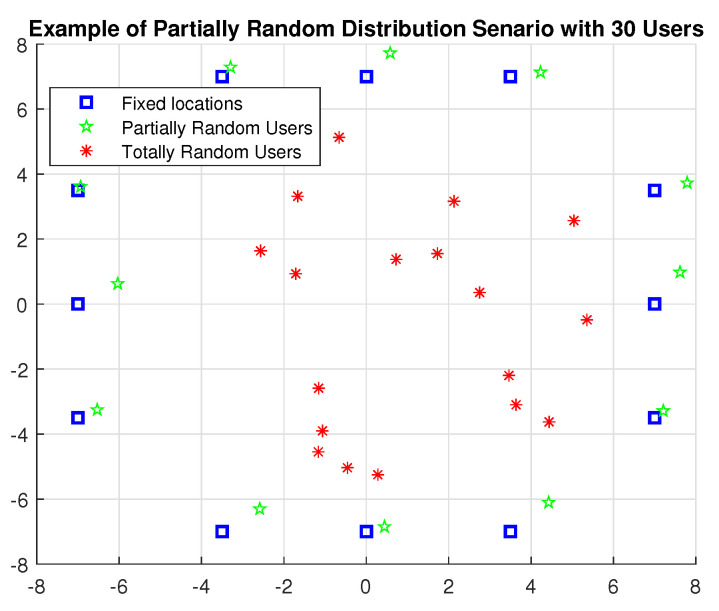
An illustration of a partially random user distribution.

The covered interior space is 16 m × 16 m. Assuming that there is no optical ICI, each LiFi attocell within a circle with a radius of 4 m operates in the same frequency range. There are eight meters between each pair of nearby LiFi APs. A partially random distribution (PRD) scenario essentially covers the available area [27]. To imitate people seated around offices or fixed tables, certain users in the PRD are partially bound to specific fixed places within the 0.5 m range, while the remaining users are free to roam to any location within the coverage area. The moving users are randomly dispersed and travel randomly in the random way-point paradigm, which was proposed in [41,42]. This model states that each node travels to a selected target location at a speed that is evenly distributed along the [$V_{min}$ = 0.3 m/s, $V_{max}$ = 0.7 m/s]. Once the objective has been reached, the node stops for a while before deciding on a new target to travel towards at a different speed. Figure 2 depicts examples of PRD distributions for 30 mobile users. The additional parameters are shown in Table 1.

For downlink communications, the proposed CMCD and all earlier algorithms are evaluated based on the measurements of the outage probability and feasible data rates. The user data rate for state *n* for all algorithms is calculated as follows:

(26) $$R_{\mu }^{\left(n\right)}=\frac{1}{N_{\mu }}\sum_{\alpha \in C}g_{\mu ,\alpha }^{\left(n\right)}k_{\mu ,\alpha }^{\left(n\right)}r_{\mu ,\alpha }^{\left(n\right)},$$

where $g_{\mu ,\alpha }^{\left(n\right)}$ is given in Equation (16), and Equation (19) for SOA, and other algorithms, respectively.

The outage probability for each user is given as follows:

(27) $$\Phi_{0}=\mathrm{Pr}\left(R_{\mu }^{n}<\Gamma_{0}\right),$$

where $\Gamma_{0}$ denotes a standard minimum data rate made available to users. The outage probability is computed using Monte Carlo simulations as follows:

(28) $$\Phi_{0}=\frac{\Sigma_{n}\mathrm{Number}\;\mathrm{of}\;\mathrm{Users}\;\mathrm{with}R_{\mu }^{n}<\Gamma_{0}}{\mathrm{Total}\;\mathrm{Number}\;\mathrm{of}\;\mathrm{Users}}$$

### 5.2. Correlation-Weighted Majority Voting (CWMV) Performance

**Figure 3:**
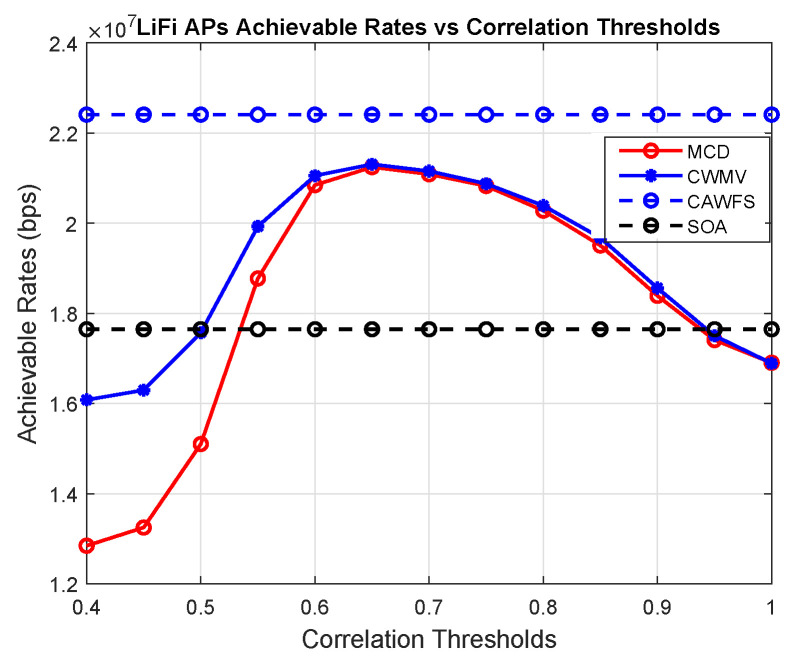
Correlation-weighted majority voting effect on the achievable data rates of the LiFi AP users.

In this subsection, the effect of the modified majority voting on the overall performance is evaluated. The conventional MCD algorithm is used with the proposed CWMV instead of the old MMV algorithm from [27]. In this part, the achievable data rates versus the correlation threshold are calculated using MCD with old MMV and MCD with the proposed CWMV, CAWFS, and SOA algorithms. The impact of the suggested CWMV algorithm on the average data rates that LiFi AP users may achieve is shown in Figure 3. The simulation findings demonstrate that, in comparison to MCD and SOA methods, implementing the suggested CWMV algorithm enhances the possible data rate performance for LiFi AP users, particularly at low correlation threshold levels.

**Figure 4:**
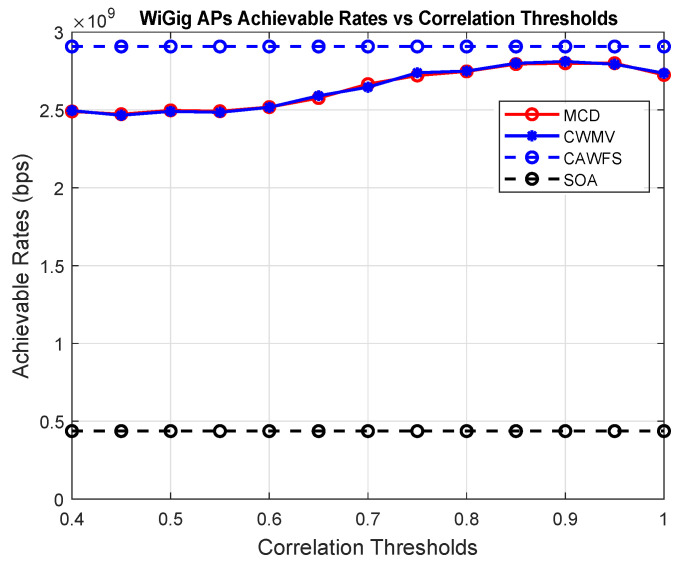
Correlation-weighted majority voting effect on the achievable data rates of the WiGig AP users.

Figure 4 shows the effect of the proposed CWMV algorithm on the achievable data rates of the WiGig AP users. The simulation results show that using the proposed CWMV algorithm has no effect on the achievable data rate performance for WiGig AP users in comparison to MCD and SOA methods.

**Figure 5:**
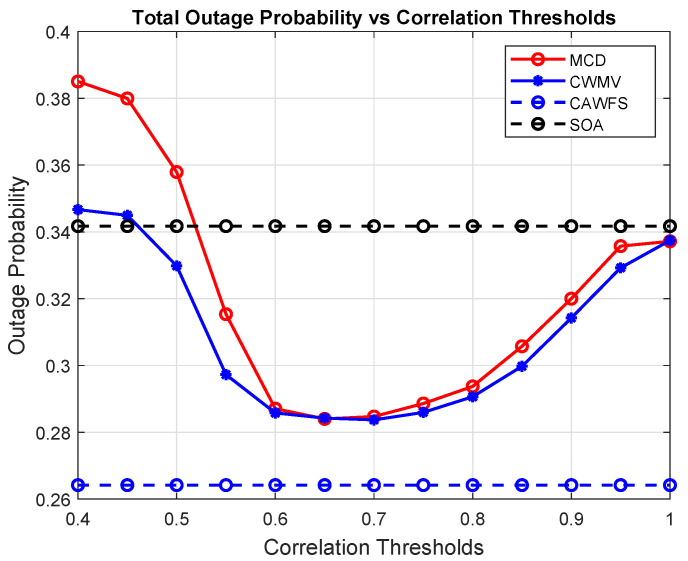
Correlation-weighted majority voting effect on the outage probability of all users.

Figure 5 shows the effect of the proposed CWMV algorithm on the outage probability of all users. The simulation results show that using the proposed CWMV algorithm improves the outage probability performance for all mobile users in comparison to MCD and SOA algorithms, especially with low-correlation threshold values.

### 5.3. Reference Mode Effect

In Algorithm 6, it is stated that the performance optimization of LiFi and/or WiGig users may be used as a reference mode to determine the condition for invoking the CAWFS algorithm. In this subsection, the effect of the chosen reference mode—LiFi and/or WiGig users’ performance optimization—on the overall performance is evaluated. In this part, the achievable data rates versus the correlation threshold are calculated using MCD, conditional MCD with all users (i.e., LiFi + WiGig) reference mode, conditional MCD with LiFi users reference mode, and conditional MCD with WiGig users reference mode, CAWFS, and SOA algorithms. Here, to test the effect of reference mode only, the MMV, not CWMV, algorithm is used.

**Figure 6:**
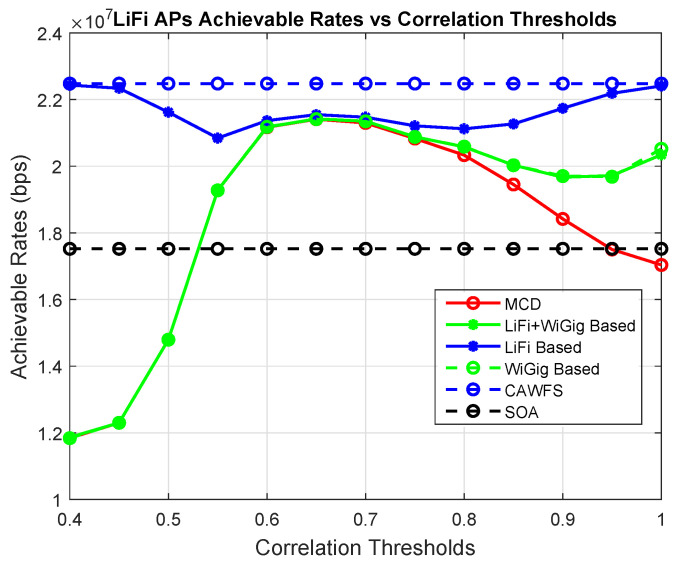
Reference mode effect on the achievable data rates of the LiFi AP users.

Figure 6 shows the effect of the chosen reference mode on the achievable data rates of the LiFi AP users. The simulation results show that using the LiFi users’ performance as a reference mode provides the best achievable data rates for LiFi users in comparison with using other reference modes.

**Figure 7:**
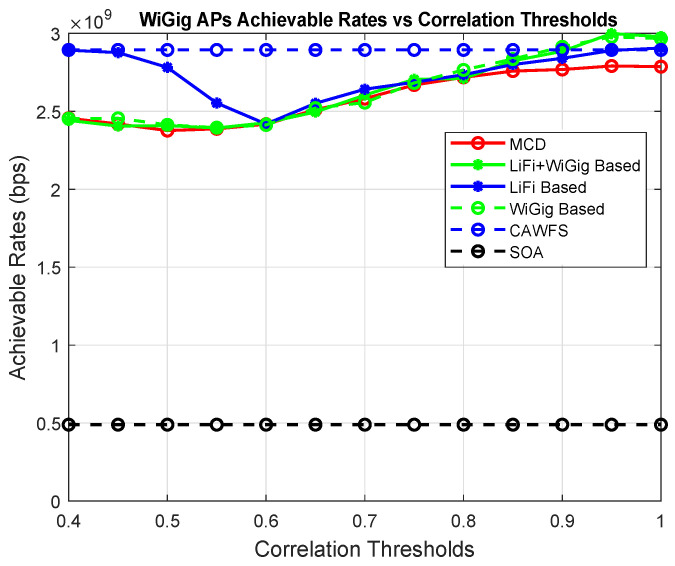
Reference mode effect on the achievable data rates of the WiGig AP users.

Figure 7 shows the effect of the chosen reference mode on the achievable data rates of the WiGig AP users. The simulation results show that using the LiFi users’ performance as a reference mode provides the best achievable data rates for WiGig users in comparison with using other reference modes.

**Figure 8:**
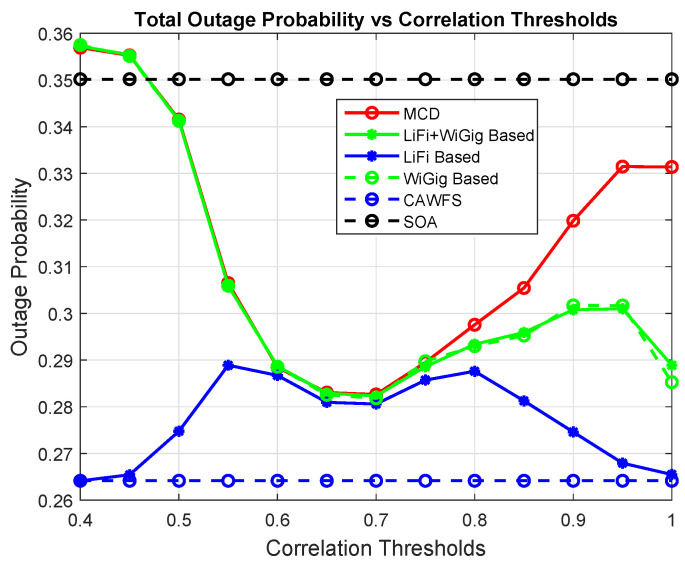
Reference mode effect on the outage probability of all users.

Figure 8 shows the effect of the chosen reference mode on the outage probability for all LiFi and WiGig users. The simulation results show that using the LiFi users’ performance as a reference mode provides the best outage probability for all users when compared with other reference modes.

### 5.4. Update The DDR Record Effect

According to the proposed CMCD algorithm in Section 4, after APA decision calculations using the MCD or CAWFS algorithm, the DDR record will be updated by adding the new distribution and its corresponding APA decisions. This subsection assesses how the DDR update procedure affects the overall performance. This section uses the MCD, conditional MCD without DDR updating (ConMCD), conditional MCD with DDR updating (ConUpMCD), CAWFS, and SOA algorithms to determine the possible data rates vs. the correlation threshold. Here, to test the effect of the DDR updating process only, the MMV, not CWMV, algorithm is used. Also, the LiFi users’ performance as a reference mode is deployed.

**Figure 9:**
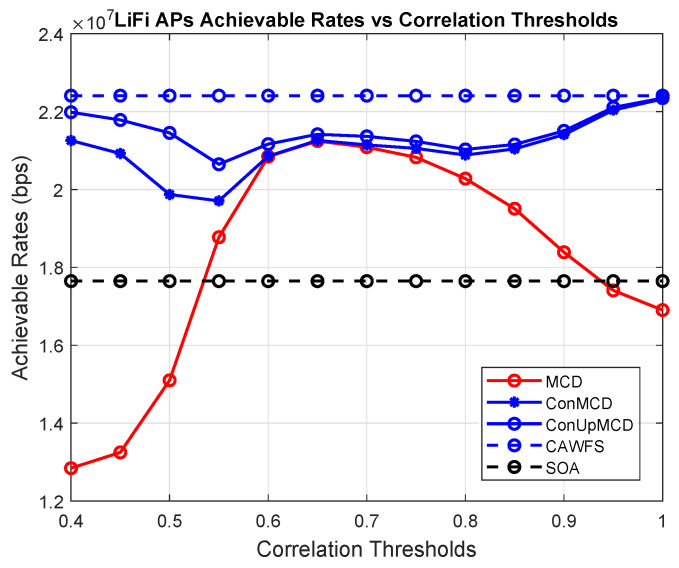
Conditional and update DDR record effect on the achievable data rates of the LiFi AP users.

Figure 9 shows the DDR updating process effect on the achievable data rates of the LiFi AP users. The simulation findings demonstrate that, when compared to MCD, ConMCD, and SOA algorithms, the ConUpMCD algorithm provides the best possible data rates for LiFi users.

**Figure 10:**
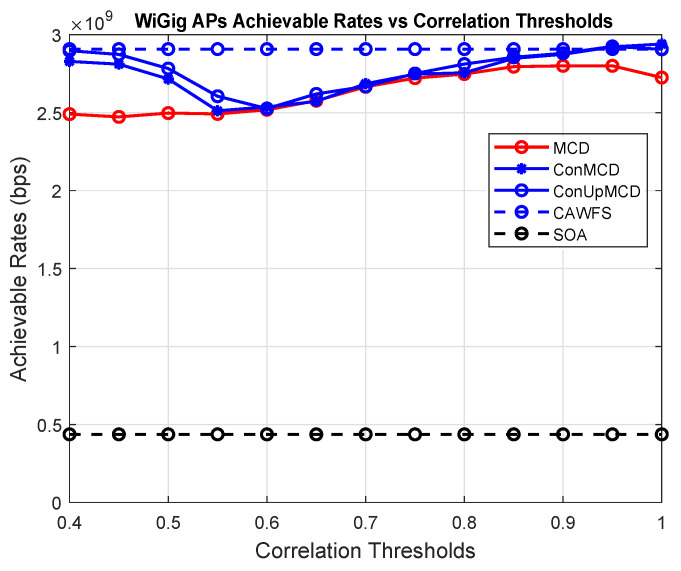
Conditional and updated DDR record effect on the achievable data rates of the WiGig AP users.

The impact of the DDR update procedure on the maximum data rates that WiGig AP users may achieve is depicted in Figure 10. The simulation results show that the ConUpMCD algorithm offers the greatest data rates for WiGig users when compared to the MCD, ConMCD, and SOA algorithms.

**Figure 11:**
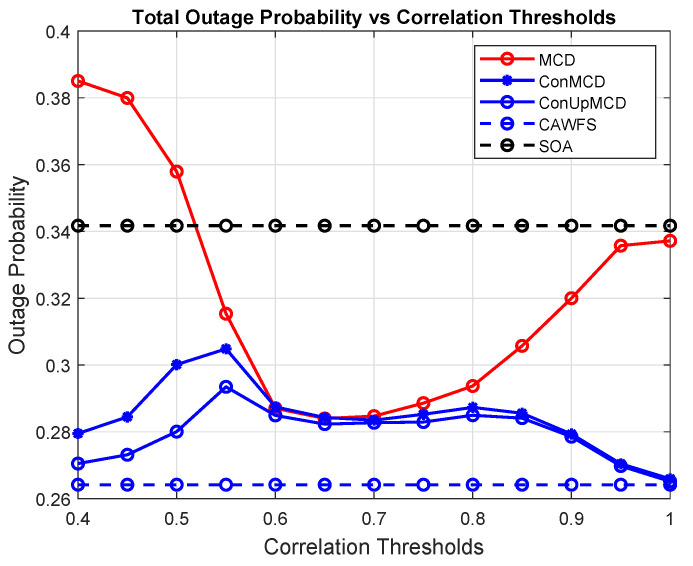
Conditional and updated DDR record effects on the outage probability of all users.

The DDR upgrading process’s impact on the overall users’ outage probabilities is depicted in Figure 11. According to the simulation results, the ConUpMCD algorithm has the highest outage probability when compared to MCD, ConMCD, and SOA algorithms.

### 5.5. The Proposed CMCD Algorithm Performance

The effectiveness of the proposed CMCD algorithm, including the CWMV and DDR record updating processes, is assessed in this subsection. This part compares the proposed CMCD algorithm to the MCD, CAWFS, and SOA algorithms by calculating the achievable data rates and outage probability vs. the correlation threshold.

**Figure 12:**
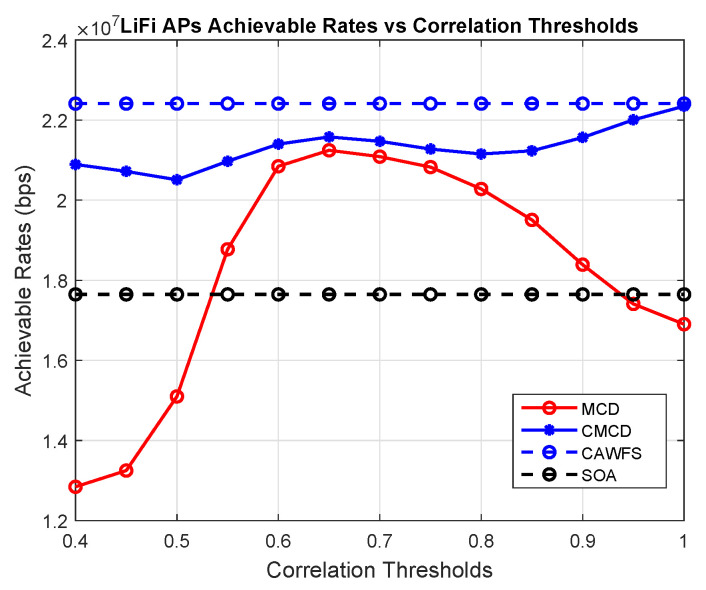
Effect of the proposed CMCD algorithm on the achievable data rates of LiFi AP users.

The impact of the suggested CMCD algorithm on the maximum data rates that LiFi AP users may achieve is shown in Figure 12. The simulation results show that the CMCD algorithm offers LiFi users the highest achievable data rates when compared to the MCD and SOA algorithms.

**Figure 13:**
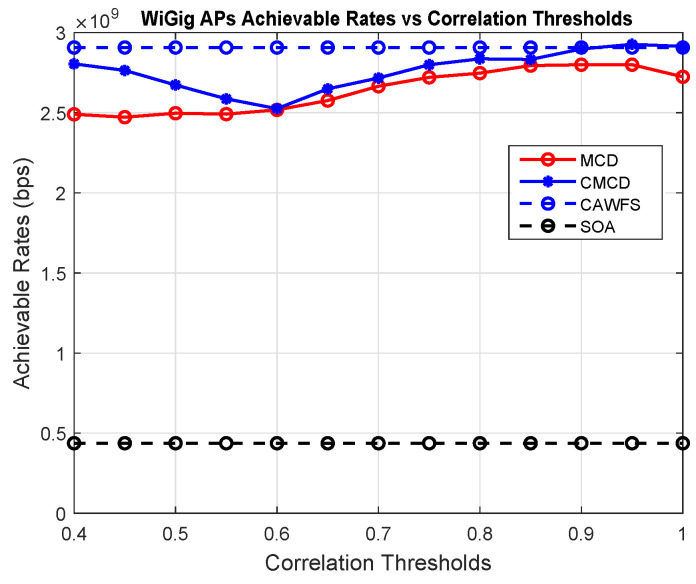
Effect of the proposed CMCD algorithm on the achievable data rates of WiGig AP users.

The impact of the suggested CMCD algorithm on the WiGig AP users’ achievable data rates is seen in Figure 13. The simulation results show that the CMCD algorithm delivers the best data rates for WiGig users when compared to MCD and SOA algorithms.

**Figure 14:**
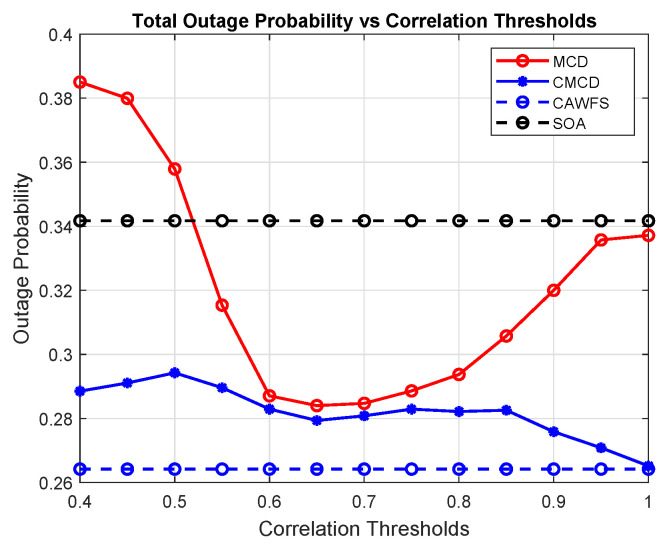
Effect of the proposed CMCD algorithm on the outage probability of all users.

The proposed CMCD algorithm’s impact on all users’ outage probability is depicted in Figure 14. The simulation results show that the CMCD algorithm offers the best outage probabilities for all users when compared to MCD and SOA methods.

### 5.6. Tolerance Factor Effect

In the first step of the proposed CMCD method in Section 4, the APA decision is made using the low-complexity MCD algorithm, and the average data rate performances of the LiFi and WiGig APs assigned users, $R_{l}$ and $R_{w}$, respectively, are calculated. These decisions will be confirmed if the APA decisions exhibit a satisfactory performance when compared to a certain threshold $\eta_{th}$. On the other side, the APA decisions utilizing the more complex but accurate CAWFS algorithm are computed if the given performance is below the threshold $\eta_{th}$. The number of times the proposed CMCD algorithm will invoke the CAWFS method, which depends on the calling threshold $\eta_{th}$, determines the comparative complexity, specified by the complexity reduction ratio CRR from Equation (21), as well as the performance quality. The simulation findings in the Section 5.3 show that the performance of the LiFi users, $R_{l}$, used as a reference mode, offers the best performance for all users. So, the calling threshold $\eta_{th}$ may be related $R_{l}$ through the tolerance factor ρ:

(29) $$\eta_{th}=\rho \times R_{l}$$

**Figure 15:**
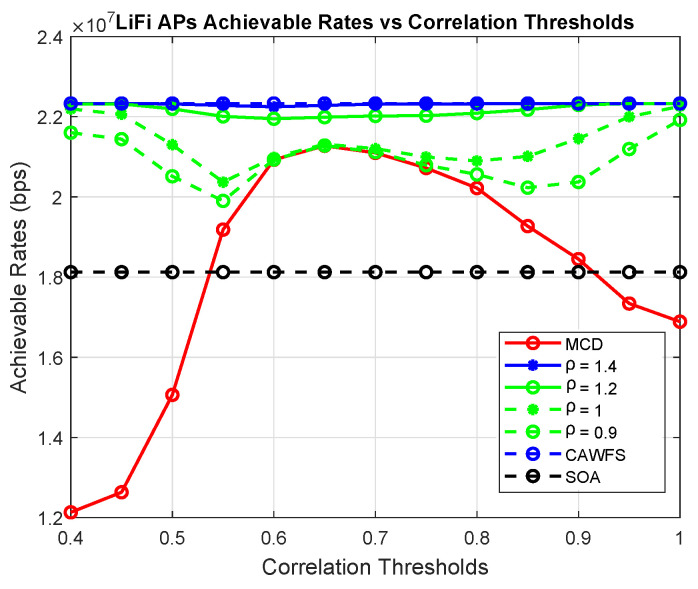
Tolerance factor effect on the achievable data rates of the LiFi AP users.

**Figure 16:**
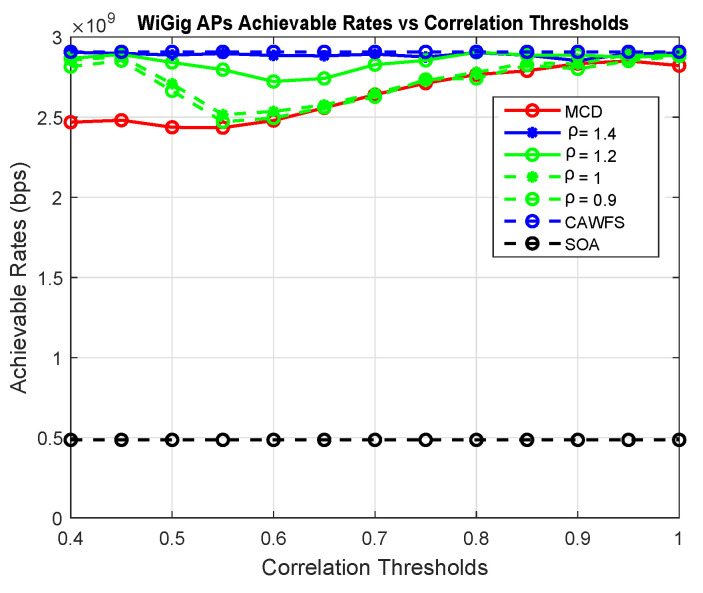
Tolerance factor effect on the achievable data rates of the WiGig AP users.

This subsection assesses how the tolerance factor ρ affects the overall performance. Using MCD, the suggested CMCD with various tolerance factors, CAWFS, and SOA algorithms, the achievable data rates and outage probability vs. the correlation threshold are computed. The effect of the tolerance factor on potential data rates for LiFi and WiGig AP users is seen in Figure 15 and Figure 16, respectively. In comparison to the smaller tolerance factors, ρ = 1.2, 1, or 0.9, the simulation results demonstrate that the higher tolerance factor, ρ=1.4, gives users the greatest achievable data rates.

**Figure 17:**
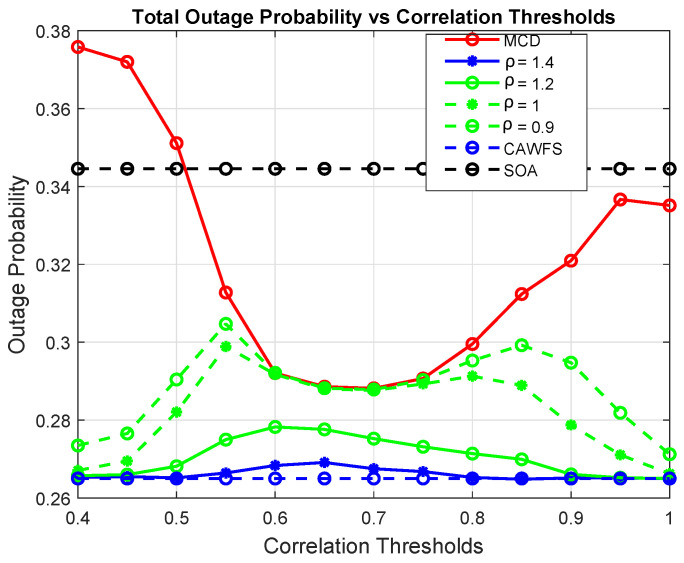
Tolerance factor effect on the outage probability of all users.

Figure 17 depicts how the tolerance factor affects all users’ outage probabilities. The simulation findings show that the greater tolerance factor, ρ=1.4, offers users the lowest outage possibilities in contrast to the lower tolerance factors, ρ = 1.2, 1, or 0.9.

**Figure 18:**
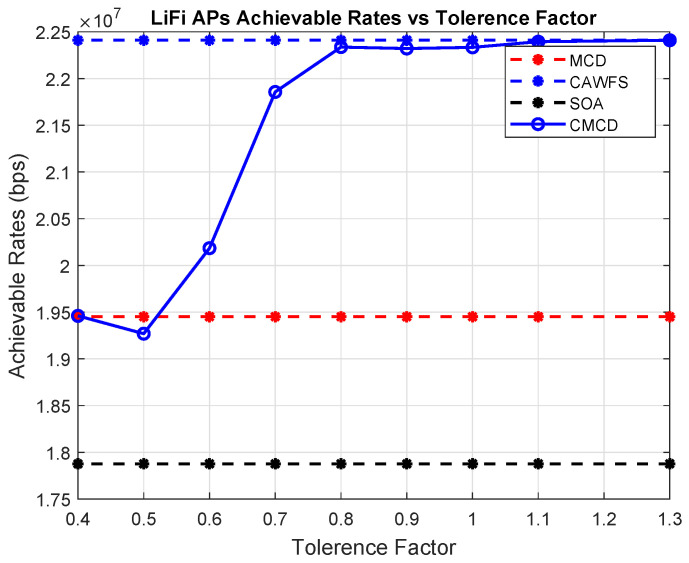
The achievable data rates of the LiFi AP users vs. various tolerance factors.

**Figure 19:**
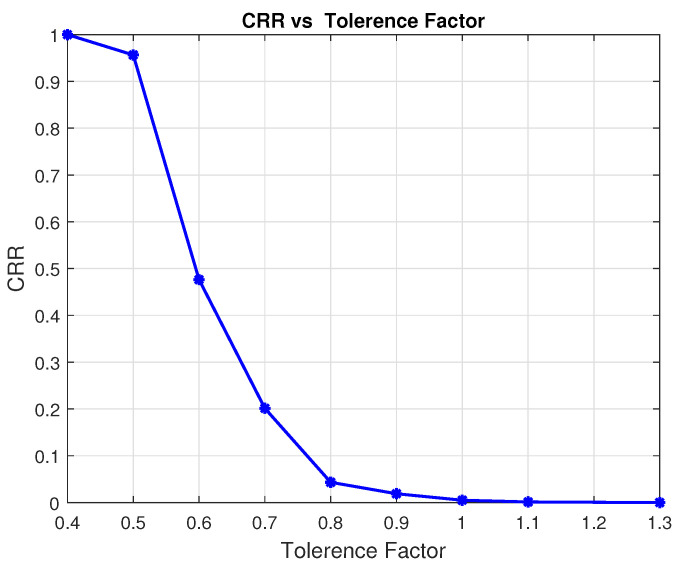
Tolerance factor effect on the CRR.

Equation (29) states that raising the tolerance factor ρ will raise the calling threshold $\eta_{th}$. As a result, the CAWFS performance will be offered, and overall performance will be enhanced. However, this will result in a decrease in the CRR value and an increase in the overall complexity of the proposed CMCD algorithm. To demonstrate this performance-complexity trade-off, Figure 18 and Figure 19 illustrate the achievable data rates for LiFi AP users and the complexity reduction ratios CRR vs. different tolerance factors ρ, respectively.

The effect of ρ values on the complexity reduction ratios percentage CRR% is shown in Table 2. For high tolerance factor, ρ=1.3, the performance of the low-complexity MCD algorithm will not be acceptable, and the high-complexity CAWFS algorithm will be completely called. In this situation, no complexity reduction is achieved, CRR%=0%. On the other hand, for low tolerance factor, ρ=0.4, the performance of the low-complexity MCD algorithm will be completely acceptable, and the high-complexity CAWFS algorithm will be never called. In this situation, full complexity reduction is achieved, CRR%=100%.

## 6. Conclusions

This article proposes the conditional most-correlated distribution (CMCD) load-balancing strategy. Depending on the achievable data rate performance, the suggested strategy offers a flexible and simple technique to switch between the simpler MCD algorithm and the more complex but more accurate CAWFS algorithm. The proposed CMCD algorithm provides a clear performance–complexity trade-off, which could be simply controlled by choosing the suitable tolerance factor value. Additionally, the correlation-weighted majority voting (CWMV) algorithm is also proposed. This algorithm seeks to improve the performance of the previously proposed majority voting algorithm by adjusting the weight of each vote in accordance with the correlation coefficients between their respective distribution vectors and the incoming distribution vector. In the simulation section, each component of the proposed algorithm is separately evaluated and the improvements in both achievable data rates and outage probabilities of all users are shown.

## Figures and Tables

**Table 1:** The simulation parameters.

Parameter	Value
LiFi subnetwork’s configuration settings	
The LiFi attocell’s radius	4 m
The covered room’s height	2.3 m
Energy conversion from electrical to optical, ι	1
The LiFi AP’s transmitted optical power, Pt	10 W
Base-band bandwidth of LED lights, B	20 MHz
The photo-detector’s (PD) physical area, $A_{p}$	1 cm^2^
Angle of half-intensity radiation, $\theta_{1/2}$	60 deg
The gain of the optical filter, $T_{s}\left(\theta \right)$	1.0
The receiver’s FoV semi-angle, $\Theta_{F}$	60 deg
Index of refractive, χ	1.5
Opto-to-electric conversion efficiency, κ	0.53 A/W
Spectral density of noise power, $N_{0}$	$10^{-19}$ A^2^/Hz
The time frame for resource distribution, $T_{p}$	500 ms
WiGig subnetwork’s configuration settings	
How many antennas are on the base station side, $N_{BS}$	25
How many antennas are on the mobile user side, $N_{MS}$	9
Maximum of WiGig AP allocated users, $N_{max}$	6
Channel SNR,	0 dB
Number of paths, *l*	1

**Table 2:** Tolerance factor effect on the CRR.

ρ	1.30	1.10	1.00	0.90	0.80	0.70	0.60	0.50	0.40
CRR%	0.00	0.09	0.52	1.40	4.21	16.63	48.54	93.45	100.00

## Data Availability

Data sharing is not applicable to this article.
